# Supplementary material for: Hybrid Models and Biological Model Reduction with PyDSTool
Source: PLoS Comput Biol. 2012 Aug 9;8(8):e1002628. doi: 10.1371/journal.pcbi.1002628 (PMC3415397; doi:10.1371/journal.pcbi.1002628)
Supplement: Text S4 — Complete source code for the PyDSTool package (version 0.88.120504). Includes API documentation and help files linking to web pages. This file is identical to the current public release on Sourceforge.net. (ZIP) [file pcbi.1002628.s004.zip › PyDSTool/html/PyDSTool.Toolbox.ParamEst-module.html]

xml version="1.0" encoding="ascii"?


PyDSTool.Toolbox.ParamEst


| Home | Trees | Indices | Help | PyDSTool |
| --- | --- | --- | --- | --- |

Package PyDSTool ::
Package Toolbox ::
Module ParamEst

[hide private]

[frames] | no frames]

# Module ParamEst

source code

Parameter estimation classes for ODEs.

Robert Clewley.


|  |  |
| --- | --- |
| Classes | [hide private] |

Converged

ConstraintFail

residual\_fn\_context

residual\_fn\_context\_1D

L2\_feature\_1D  
Use with scalar optimizers such as BoundMin

L2\_feature

ParamEst  
General-purpose parameter estimation class.

LMpest  
Unconstrained least-squares parameter and initial condition
optimizer for n-dimensional DS trajectories.

BoundMin  
Bounded minimization parameter and initial condition optimizer for
one-dimensional DS trajectories.


|  |  |
| --- | --- |
| Functions | [hide private] |

|  |  |
| --- | --- |
| do\_2Dstep(fun, p, dirn, maxsteps, stepsize, atol, i0, orig\_res, orig\_dirn, all\_records)  Residual vector continuation step in 2D parameter space. | source code |

|  |  |
| --- | --- |
| do\_2Ddirn(fun, p0, dirn, maxsteps, stepsize, atol, orig\_res, orig\_dirn, all\_records)  Residual vector continuation in a single neutral direction in 2D parameter space, given by dirn = +1/-1 from point p0. | source code |

|  |  |
| --- | --- |
| ctn\_residual\_info(recs, do\_plot=False)  Temporary helper function for use with continuation functions. | source code |

|  |  |
| --- | --- |
| sweep1D(fun, interval, resolution) | source code |

|  |  |
| --- | --- |
| grad\_from\_psens(psens, pest) | source code |

|  |  |
| --- | --- |
| filter\_feats(parname, feat\_sens)  Filter features whose residual vectors show a \*net\* increase (dirn=1) or decrease (dirn=-1) as one parameter is varied. | source code |

|  |  |
| --- | --- |
| filter\_pars(mi\_feat, feat\_sens)  For a given (model interface, feature) pair, find all parameters that change the feature's net residual in the same direction, or not at all. | source code |

|  |  |
| --- | --- |
| \_present\_and\_sensitive(xsf, L, thresh, rejected, neutral)  Helper function to return whether x is present in list L with an associated sensitivity s larger than thresh, where L is made up of (y, ysens) pairs, provided the sensitivities are larger than threshold. | source code |

|  |  |
| --- | --- |
| \_makeUnique(L) | source code |

|  |  |
| --- | --- |
| pp(l)  List pretty printer | source code |

|  |  |
| --- | --- |
| select\_pars\_for\_features(desired\_feats, feat\_sens, deltas, neg\_tol=0, pos\_tol=0.001, method=`'``RecursiveBacktrackingSolver``'`, forwardCheck=True, verbose=False)  Default tol > 0 in case there are undifferentiable features that will never lead to satisfaction of constraints with tol=0. | source code |

|  |  |
| --- | --- |
| norm\_D\_sum(D\_sum)  Normalize a D\_sum by the elements for each feature by smallest absolute size (that element becomes 1 in norm). | source code |

|  |  |
| --- | --- |
| filter\_iface(psens, iface) | source code |

|  |  |
| --- | --- |
| organize\_feature\_sens(feat\_sens, discrete\_feats=None) | source code |

|  |  |
| --- | --- |
| make\_opt(pnames, resfnclass, model, context, parscales=None, parseps=None, parstep=None, parlinesearch=None, stopcriterion=None, grad\_ratio\_tol=10, use\_filter=False, verbose\_level=2)  Create a ParamEst manager object and an instance of an optimizer from the Toolbox.optimize sub-package, returned as a pair. | source code |

|  |  |
| --- | --- |
| restrict\_opt(pest, feat\_list, opt, pars=None)  Restrict parameter estimation to certain features and parameters. | source code |

|  |  |
| --- | --- |
| get\_slope\_info(x, lookahead=1, prec=0.001, default=1)  DEPRECATED. | source code |

|  |  |
| --- | --- |
| get\_extrema(x, t, tmin, tmax, coords, per, pertol\_frac, lookahead, lookahead\_tol, fit\_fn\_class=None, verbose=False)  DEPRECATED. | source code |

|  |  |
| --- | --- |
| get\_extrema\_from\_events(gen, coords, tmin, tmax, per, pertol\_frac, verbose=False)  Helper function for qualitative fitting of extrema using least squares. | source code |

|  |  |
| --- | --- |
| compare\_data\_from\_events(gen, coords, traj, tmesh, data\_mins\_t, data\_maxs\_t, data\_mins\_v, data\_maxs\_v, num\_expected\_mins, num\_expected\_maxs, tdetect, per, pertolfrac, verbose=False) | source code |


|  |  |
| --- | --- |
| Variables | [hide private] |

\_seq\_types = `(<type 'list'>, <type 'tuple'>, <type 'numpy.ndar...`

Tester

test

power = `<ufunc 'power'>`

sign = `<ufunc 'sign'>`

HAVE\_PSYCO = `True`

\_pest\_classes = `['ParamEst', 'LMpest', 'BoundMin', 'residual_f...`

\_deprecated\_functions = `['get_slope_info', 'get_extrema', 'com...`

\_ctn\_functions = `['do_2Dstep', 'do_2Ddirn', 'ctn_residual_info']`

\_generic\_opt = `['make_opt', 'restrict_opt']`

\_utils = `['sweep1D', 'filter_feats', 'filter_pars', 'select_pa...`

\_errors = `['Converged', 'ConstraintFail']`

solver\_lookup = `{'BacktrackingSolver': <class 'constraint.Back...`

rout = `redirc.Redirector(redirc.STDOUT)`

rerr = `redirc.Redirector(redirc.STDERR)`

res\_fn\_lookup = `{<class 'PyDSTool.Toolbox.ParamEst.LMpest'>: <...`


|  |  |
| --- | --- |
| Function Details | [hide private] |

|  |  |
| --- | --- |
| do\_2Dstep(fun, p, dirn, maxsteps, stepsize, atol, i0, orig\_res, orig\_dirn, all\_records) | source code |

Residual vector continuation step in 2D parameter space.

orig\_dirn corresponds to direction of positive dirn, in case when
re-calculating gradient the sign flips

|  |  |
| --- | --- |
| do\_2Ddirn(fun, p0, dirn, maxsteps, stepsize, atol, orig\_res, orig\_dirn, all\_records) | source code |

Residual vector continuation in a single neutral direction in 2D
parameter space, given by dirn = +1/-1 from point p0.

maxsteps is \*per\* direction

|  |  |
| --- | --- |
| filter\_feats(parname, feat\_sens) | source code |

Filter features whose residual vectors show a \*net\* increase (dirn=1)
or decrease (dirn=-1) as one parameter is varied. Provided the
sensitivities were measured appropriately, dirn=0 will select any
non-smoothly changing features (e.g. discrete-valued).

feat\_sens is a dictionary of feature sensitivities keyed by parameter
name, e.g. as returned by the ParamEst.par\_sensitivity method.

Returns a list of ((model interface, feature), sensitivity) pairs,
where the feature belongs to the model interface (in case of duplication
in multiple interfaces), and the sensitivity is the absolute value of the
net increase/decrease. The lists are ordered by decreasing magnitude of
sensitivity.

Definition of net increase: e.g. if sensitivity for a given feature
with a 3-vector residual is [-0.1 0.4 1.5] then the sum is +1.8 and will
be selected for the 'increasing' direction.

|  |  |
| --- | --- |
| filter\_pars(mi\_feat, feat\_sens) | source code |

For a given (model interface, feature) pair, find all parameters that
change the feature's net residual in the same direction, or not at all.
(Provided the sensitivities were measured appropriately, this will select
any non-smoothly changing features (e.g. discrete-valued)).

feat\_sens is a dictionary of feature sensitivities keyed by parameter
name, e.g. as returned by the ParamEst.par\_sensitivity method.

Returns a triple of lists of (parameter names, sensitivity) pairs:
increasing, decreasing, and neutral. The lists are ordered by decreasing
magnitude of sensitivity.

|  |  |
| --- | --- |
| \_present\_and\_sensitive(xsf, L, thresh, rejected, neutral) | source code |

Helper function to return whether x is present in list L with an
associated sensitivity s larger than thresh, where L is made up of (y,
ysens) pairs, provided the sensitivities are larger than threshold.

xsf is the triple (x, xsens, feature). rejected and neutral arguments
should be lists to store the new rejected and neutral items.

|  |  |
| --- | --- |
| select\_pars\_for\_features(desired\_feats, feat\_sens, deltas, neg\_tol=0, pos\_tol=0.001, method=`'``RecursiveBacktrackingSolver``'`, forwardCheck=True, verbose=False) | source code |

Default tol > 0 in case there are undifferentiable features that
will never lead to satisfaction of constraints with tol=0.

Returns a problem object and the by-parameter sensitivity dictionary
of derivatives, D.

|  |  |
| --- | --- |
| norm\_D\_sum(D\_sum) | source code |

Normalize a D\_sum by the elements for each feature by smallest
absolute size (that element becomes 1 in norm). For unweighted feature
sensitivities, or else it unweights weighted ones.

|  |  |
| --- | --- |
| make\_opt(pnames, resfnclass, model, context, parscales=None, parseps=None, parstep=None, parlinesearch=None, stopcriterion=None, grad\_ratio\_tol=10, use\_filter=False, verbose\_level=2) | source code |

```
Create a ParamEst manager object and an instance of an optimizer from the
Toolbox.optimize sub-package, returned as a pair.

Inputs:

pnames:     list of free parameters in the model
resfnclass: residual function class (e.g. residual_fn_context_1D exported
            from this module)
model:      the model to optimize, of type Model (not a Generator)
context:    the context object that defines the objective function
            criteria via "model interfaces" and their features, etc.
parscales:  for models that do not have parameters varying over similar
            scales, this dictionary defines what "O(1)" change in dynamics
            refers to for each parameter. E.g. a parameter that must change by
            several thousand in order to make an O(1) change in model output
            can have its scale set to 1000. This will also be the maximum
            step size in that direction for the Scaled Line Search method, if used.
            Defaults to 10*parseps for each parameter.
parseps:    dictionary to indicate what change in parameter value to use for
            forward finite differencing, for reasons similar to those given in
            description of the parscales argument. Default is 1e-7 for each parameter.
parstep:    choice of optimization algorithm stepper, defaults to
            conjugate gradient step.CWConjugateGradientStep.
parlinesearch:  choice of line search method, defaults to scaled
                line search method line_search.ScaledLineSearch.
stopcriterion:  choice of stop criteria for the optimization iterations. Defaults to
                ftol=1e-7, gtol=1e-7, iterations_max=200.
grad_ratio_tol: For residual functions with poor smoothness in some directions,
                this parameter (default = 10) prevents those directions being used
                for gradient information if the ratio of residual values found during
                finite differencing is greater in magnitude than this tolerance value.
                (Experimental option only -- set very large, e.g. 1e6 to switch off).
use_filter:     activate use of filtering out largest directions of gradients that may
                be unreliable. Default is False. (Experimental option only).
verbose_level:  Default to 2 (high verbosity).
```

|  |  |
| --- | --- |
| restrict\_opt(pest, feat\_list, opt, pars=None) | source code |

Restrict parameter estimation to certain features and parameters.

If pars is None (default) then all free parameters of pest are
used.

|  |  |
| --- | --- |
| get\_slope\_info(x, lookahead=1, prec=0.001, default=1) | source code |

DEPRECATED. Use features - they work more efficiently and robustly.
e.g. see Toolbox/neuro\_data.py

Helper function for qualitative fitting.

Local slope information about data array x. Values of 1 in the return
array indicate increasing slopes over a local extent given by the the
lookahead argument, whereas 0 indicates non-increasing slopes.

The default value specifies the value taken by the returned array in
the indices from len(x)-lookahead to len(x).

|  |  |
| --- | --- |
| get\_extrema(x, t, tmin, tmax, coords, per, pertol\_frac, lookahead, lookahead\_tol, fit\_fn\_class=None, verbose=False) | source code |

```
DEPRECATED. Use features - they work more efficiently and robustly.
e.g. see Toolbox/neuro_data.py

Helper function for qualitative fitting.

per is an estimate of the period, per <= tmax.
pertol_frac is fraction of period used as tolerance for finding extrema.
fit_fn_class switches on interpolation of extremum by the fitting of a
      local function (uses least squares criterion) - specify a sub-class
      of fit_function (default None).
```

|  |  |
| --- | --- |
| get\_extrema\_from\_events(gen, coords, tmin, tmax, per, pertol\_frac, verbose=False) | source code |

Helper function for qualitative fitting of extrema using least
squares. This function returns the variable values at the extrema, unlike
the related function get\_extrema (for data).

per is an estimate of the period, per <= tmax. pertol\_frac is
fraction of period used as tolerance for finding extrema.

  


|  |  |
| --- | --- |
| Variables Details | [hide private] |

### \_seq\_types

Value:
:   ```
    (<type 'list'>, <type 'tuple'>, <type 'numpy.ndarray'>)
    ```

### \_pest\_classes

Value:
:   ```
    ['ParamEst',
     'LMpest',
     'BoundMin',
     'residual_fn_context',
     'residual_fn_context_1D',
     'L2_feature',
     'L2_feature_1D']
    ```

### \_deprecated\_functions

Value:
:   ```
    ['get_slope_info',
     'get_extrema',
     'compare_data_from_events',
     'get_extrema_from_events']
    ```

### \_utils

Value:
:   ```
    ['sweep1D',
     'filter_feats',
     'filter_pars',
     'select_pars_for_features',
     'grad_from_psens',
     'norm_D_sum',
     'filter_iface',
     'organize_feature_sens']
    ```

### solver\_lookup

Value:
:   ```
    {'BacktrackingSolver': <class 'constraint.BacktrackingSolver'>,
     'MinConflictsSolver': <class 'constraint.MinConflictsSolver'>,
     'RecursiveBacktrackingSolver': <class 'constraint.RecursiveBacktracki
    ngSolver'>}
    ```

### res\_fn\_lookup

Value:
:   ```
    {<class 'PyDSTool.Toolbox.ParamEst.LMpest'>: <class 'PyDSTool.Toolbox.
    ParamEst.residual_fn_context'>,
     <class 'PyDSTool.Toolbox.ParamEst.BoundMin'>: <class 'PyDSTool.Toolbo
    x.ParamEst.residual_fn_context_1D'>}
    ```

  


| Home | Trees | Indices | Help | PyDSTool |
| --- | --- | --- | --- | --- |

|  |  |
| --- | --- |
| Generated by Epydoc 3.0.1 on Fri May 4 15:24:04 2012 | http://epydoc.sourceforge.net |
